# Case Report: Two clinical cases of severe deep infiltrating endometriosis with infertility—transplantation first or surgery first? Natural cycle or artificial cycle?

**DOI:** 10.3389/fmed.2025.1725614

**Published:** 2025-12-16

**Authors:** Fei Tang, Ye He, Peipei Guo, Yue Wang, Yunyun Liu, Liting Wang, Yi Wang, Pengxiang Xie, Youyan Fang, Caihua Li, Zhaojuan Yu, Xuqing Li, Zhaolian Wei

**Affiliations:** 1 Department of Obstetrics and Gynecology, NHC Key Laboratory of Study on Abnormal Gametes and Reproductive Tract, The First Affiliated Hospital of Anhui Medical University, Hefei, Anhui, China; 2 Engineering Research Center of Biopreservation and Artificial Organs, Ministry of Education, Hefei, Anhui, China; 3 Key Laboratory of Population Health Across Life Cycle (Anhui Medical University), Ministry of Education of the People’s Republic of China, Hefei, Anhui, China; 4 Anhui Provincial Key Laboratory of Reproductive Disorders and Obstetrics and Gynaecology Diseases, Hefei, Anhui, China

**Keywords:** deep infiltrating endometriosis case report, surgery, assisted reproductive technology, embryo transfer, natural cycle

## Abstract

Deep infiltrating endometriosis (DIE) is a severe condition frequently linked to infertility, yet the optimal integration of surgery and assisted reproductive technology (ART) remains controversial. This study reports two cases of infertility secondary to severe rectal DIE that achieved live births through a tailored, sequential strategy. The management paradigm consisted of three phases: (1) fertility preservation via embryo cryopreservation, (2) definitive laparoscopic surgical resection, and (3) a natural-cycle frozen embryo transfer (NC-FET). Postoperatively, both patients demonstrated a significant decline in CA125 levels and substantial clinical improvement. The first case involved a 27-year-old with primary infertility, while the second, more complex case involved a 31-year-old with ureteral DIE and hydronephrosis, requiring multidisciplinary surgery. These cases illustrate that an integrated approach, which strategically combines preoperative fertility preservation, radical surgery to rectify pelvic anatomy, and a physiological, hormone-free embryo transfer, can optimize the likelihood of achieving pregnancy in patients with symptomatic infertility due to severe DIE.

## Introduction

Endometriosis (EMs) is a benign gynecological disease characterized by the ectopic growth of functional endometrial tissue with invasive features similar to malignancies (1, 2). It affects 10–15% of reproductive-aged women and is a major cause of chronic pain and infertility, creating a significant burden on physical health and emotional wellbeing and overall quality of life (3, 4).

Deep infiltrating EMs (DIE) is the most severe form of the condition, defined by lesions that infiltrate to a depth of at least 5 mm (5). It commonly involves structures such as the uterosacral ligaments, rectouterine pouch, vaginal fornix, rectovaginal septum, intestinal wall, bladder wall, and ureters. Invasion of these organs can result in functional impairment, severe chronic pelvic pain, and infertility (6–9). However, given the complexity and high-risk nature of radical surgery for DIE, the optimal sequence of intervention, particularly regarding whether surgery should precede or follow assisted reproductive technology (ART) in infertile patients, remains a subject of debate.

This case report details the management of two rare and complex cases of infertility associated with DIE, with the aim of providing clinical insights and proposing a potential individualized treatment strategy.

## Case series

### Case 1

**Figure 1:**
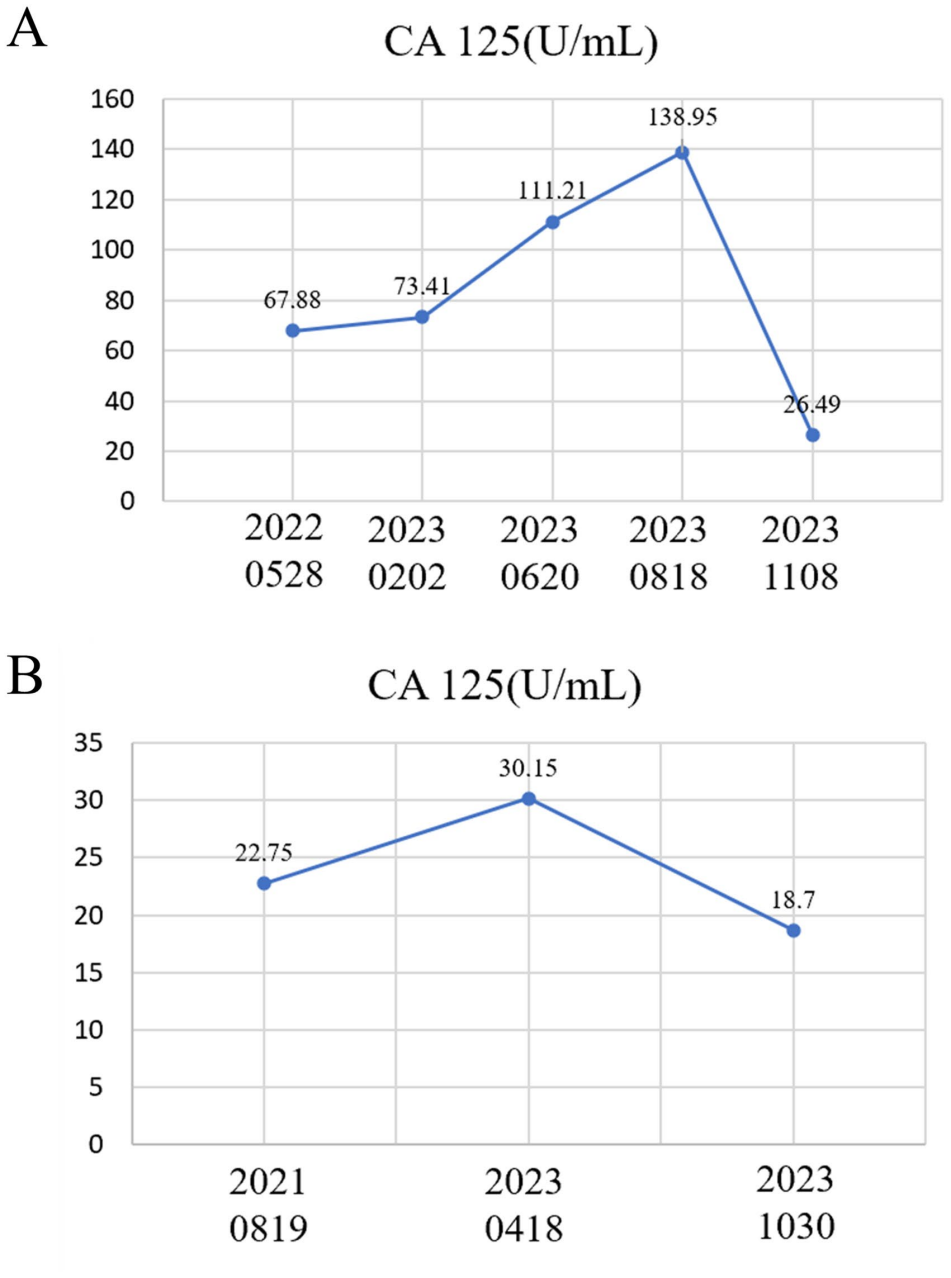
Line graph of CA125 levels at key time points during the treatment process for two patients. **(A)** In Case 1, the CA125 level exhibited a continuous upward trend from 28 May 2022 to 18 August 2023, prior to surgery. On 8 November 2023, a follow-up examination post-surgery showed that the CA125 level had decreased to 26.49 U/mL. **(B)** In Case 2, the CA125 level exhibited a continuous upward trend from 19 August 2021 to 18 April 2023, prior to surgery. A follow-up examination on 30 October 2023, post-surgery, revealed that the CA125 level had decreased to 18.7 U/mL.

In February 2023, a 27-year-old woman presented to the Reproductive Center of the First Affiliated Hospital of Anhui Medical University, complaining of primary infertility. She reported a 12-month history of prolonged menstruation and progressively worsening lower abdominal pain, with an intensity of 9 on the visual analog scale (VAS). Gynecological examination by a specialist in our team revealed a painful, nodular mass approximately 3 cm in diameter in the posterior vaginal fornix. Laboratory investigations showed an elevated serum cancer antigen 125 (CA125) of 67.88 U/mL (Figure 1A). Based on these findings, she was diagnosed with DIE and primary infertility.

**Figure 2:**
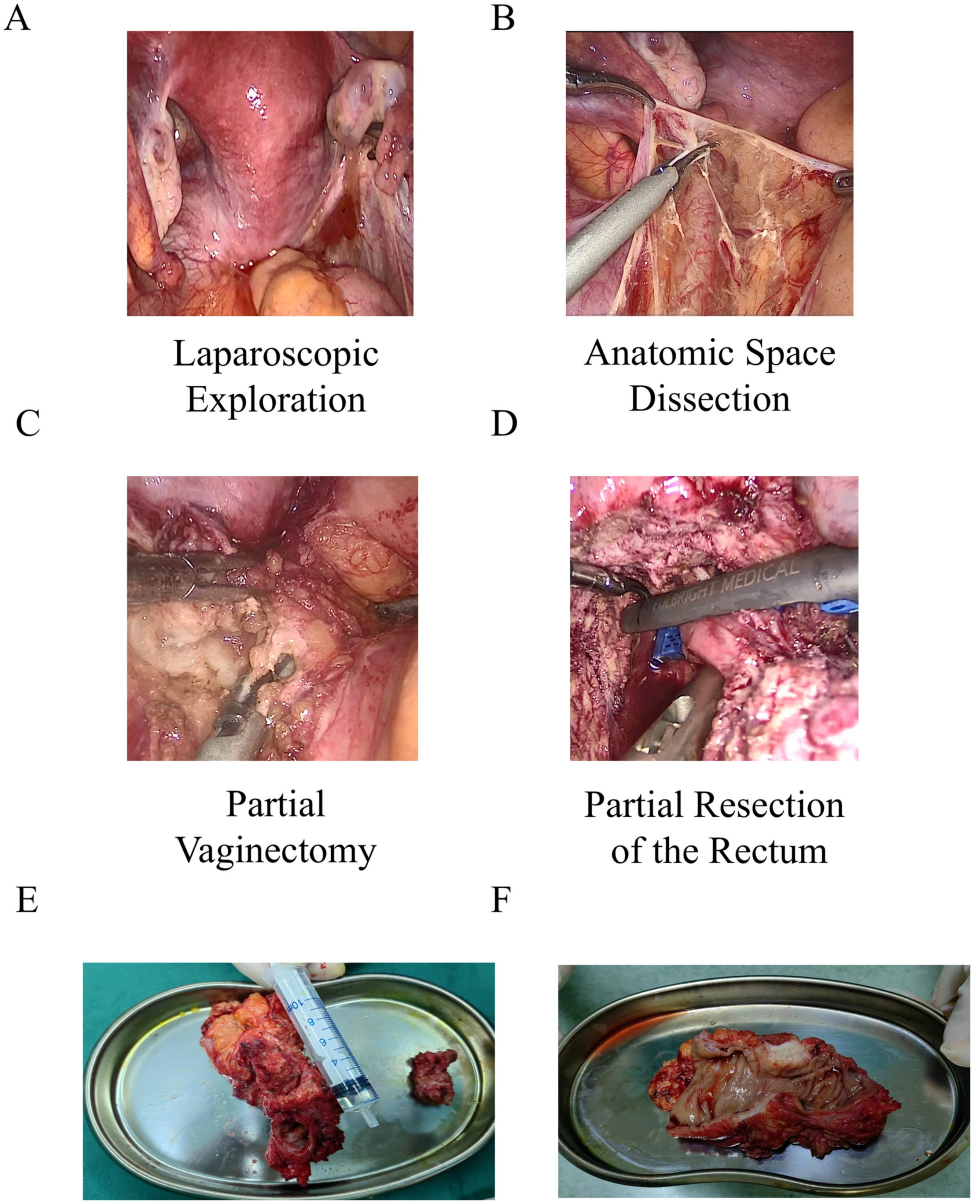
Images of the rectum and vagina resected under laparoscopy in case 1. **(A)** Laparoscopic exploration of the pelvis. **(B)** Laparoscopic dissection along anatomical planes. **(C)** Laparoscopic partial vaginectomy. **(D)** Laparoscopic partial resection of the rectum. **(E)** Segment of the rectum: approximately 8 cm in length, with an endometrial lesion on the anterior wall measuring about 5 × 3 × 3 cm, infiltrating the entire thickness of the rectal wall and reaching the mucosa. **(F)** Partial vaginal tissue: measuring 3.5 × 2 × 1 cm.

Transvaginal ultrasound indicated a normal ovarian reserve, with an antral follicle count (AFC) of 7–8 in the right ovary and 9–10 in the left. Initially, 3–4 cycles of ovulation induction were recommended; however, this approach was unsuccessful, and the patient’s symptoms of rectal pressure and difficult bowel movements worsened. A subsequent gynecological examination revealed several purple-blue nodules in the posterior vaginal fornix. The left adnexa was thickened and fixed to the pelvic wall with a firm and fixed texture. A vagino-recto-abdominal examination showed a smooth but slightly narrowed rectal mucosa, with a palpable hard mass of approximately 4 cm on the pelvic floor. Colonoscopy findings were consistent with chronic inflammation, indicating mucosal thickening over 10 cm from the anal verge, luminal narrowing, and a rough, reddened surface (Supplementary Figure S1). A multidisciplinary team meeting (MDT) recommended prompt surgical intervention for DIE. However, since infertility was this patient’s primary concern and the planned surgery was extensive with a risk of ovarian damage, a tailored strategy was adopted: ART with ovum pick-up (OPU) would be performed first, followed by scheduled DIE surgery. In July 2023, the patient underwent an *in vitro* fertilization (IVF) (antagonist protocol), resulting in the retrieval of nine oocytes and the cryopreservation of six good-quality blastocysts. This was followed by three doses of gonadotropin-releasing hormone agonist (GnRH-a). In November 2023, she underwent a 7-h laparoscopic surgery, including partial resection of the rectum and vagina and a colorectal anastomosis (Figures 2A–F). Postoperatively, she received one dose of GnRH-a. A follow-up examination showed a notable improvement in pelvic mobility and a significant alleviation of DIE-related symptoms, quantified by a reduction in the VAS pain score from 9 to 1. Postoperative transvaginal ultrasound revealed an AFC of 5–6 in each ovary.

**Figure 3:**
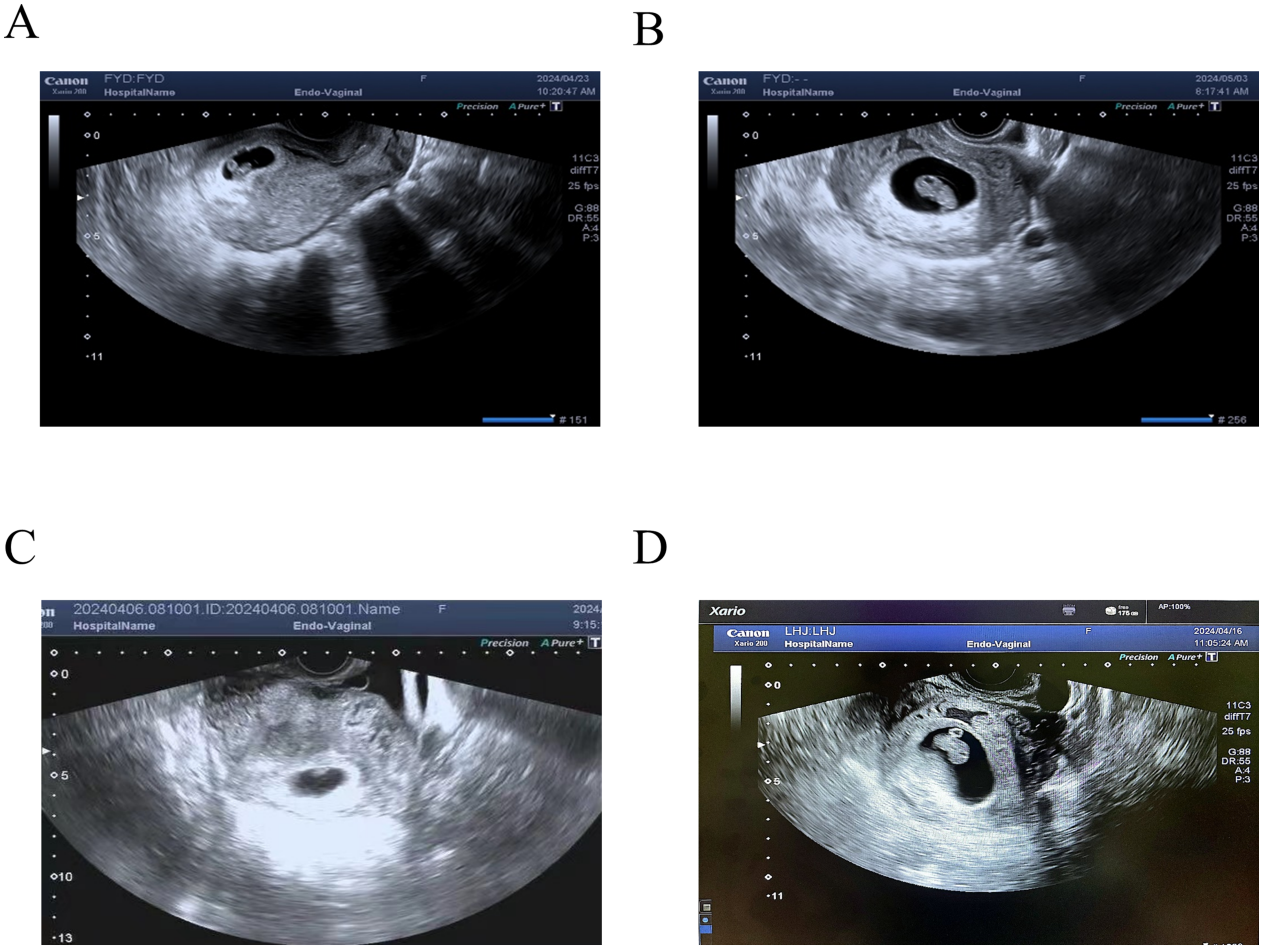
Transvaginal ultrasound images of embryonic development at 30 days and 40 days post-transfer in two patients. **(A)** The uterus is enlarged, containing a gestational sac. Inner diameter: 23 × 15 mm; yolk sac: 3.0 mm; embryo length: 9.8 mm, with visualized cardiac pulsation. There is an anechoic area measuring 20 × 10 mm surrounding the gestational sac. **(B)** The uterus is enlarged, containing a gestational sac. Inner diameter: 41 × 26 mm; yolk sac: 3 mm; embryo length: 22.4 mm, with visualized cardiac pulsation. There is an anechoic area measuring 30 × 11 mm surrounding the gestational sac. **(C)** The uterus is enlarged, containing a gestational sac. Inner diameter: 26 × 15 mm; yolk sac: 3.2 mm; embryo length: 8.5 mm, with visualized cardiac pulsation. **(D)** The uterus is enlarged, containing a gestational sac. Inner diameter: 43 × 34 mm; yolk sac: 3.6 mm; embryo length: 19.8 mm, with visualized cardiac pulsation. There is an irregular anechoic area measuring 36 × 11 mm surrounding the gestational sac.

In March 2024, a single blastocyst embryo was created following a natural cycle. The transfer resulted in a positive biochemical pregnancy, evidenced by a serum human chorionic gonadotropin (HCG) level of 1,216 mIU/mL on day 13. Ultrasound examinations at 30 and 40 days post-transfer confirmed a viable intrauterine pregnancy (Figures 3A, B). The gestational course was notable for the absence of significant DIE-related symptoms. A live male infant with an Apgar score of 10–10 at 1 and 5 min, respectively, was delivered by cesarean section at a gestational age of 38 weeks and 5 days.

### Case 2

In August 2021, a 31-year-old woman presented to our clinic with a history of primary infertility for 3 years. Her medical history included a laparoscopic right salpingo-oophorectomy performed in 2018 for an ovarian serous cystadenoma. A gynecological examination by our team revealed abnormal morphology in the posterior uterine fornix, characterized by a ring-like narrowing. Consultation with the gastrointestinal department ruled out primary intestinal disorders. Her serum CA125 level was 22.75 U/mL (Figure 1B). Pelvic ultrasound identified uterine fibroids and a normal AFC of 7–8 in the left ovary.

Given the patient’s prolonged infertility and history of right oophorectomy, priority was given to ART. In November 2021, the patient underwent an IVF cycle using a long-luteal phase protocol. 12 oocytes were retrieved, resulting in the cryopreservation of three high-quality blastocysts. Subsequently, between January and June 2022, two frozen embryo transfers were performed in artificial cycles, involving a total of three blastocysts. Unfortunately, both transfers failed to achieve a pregnancy.

In April 2023, the patient reported aggravated menstrual pain, scoring 10 on the VAS, accompanied by a narrowing of stool. Non-contrast computed tomography of the urinary system and pelvic magnetic resonance imaging revealed left hydroureteronephrosis secondary to distal ureteral stenosis. The imaging also confirmed uterine fibroids and low-density lesions on the pelvic floor. Her serum CA125 level was 30.15 U/mL (Figure 1B). A vagino-recto-abdominal examination indicated a frozen pelvis with significant rectal compression and a hardened mass on the pelvic floor, leading to a definitive diagnosis of DIE. An MDT consultation recommended a repeated IVF cycle followed by surgical intervention prior to embryo transfer. Accordingly, in May 2023, the patient underwent an IVF cycle (antagonist protocol), which resulted in the retrieval of seven oocytes and the cryopreservation of two high-quality blastocysts.

In July 2023, she underwent an extensive laparoscopic procedure at the First Affiliated Hospital of Sun Yat-sen University in Guangzhou. The surgery lasted 11 h involved resection of lesions from the ligaments and rectovaginal septum, hysteroscopy with excision of endometrial polyps, laparoscopic myomectomy, partial laparoscopic ureterectomy with ureteral-bladder anastomosis, laparoscopic adhesiolysis of surrounding ureters, insertion of ureteral stents via cystoscopy, laparoscopic pelvic, intestinal, and abdominal adhesiolysis, and partial laparoscopic resection of the rectum. Postoperatively, the patient was administered dienogest orally for 4 months. By October 2023, her serum level of CA125 had decreased to 18.7 U/mL (Figure 1B), and follow-up imaging confirmed the resolution of hydronephrosis. Her bowel function normalized, and a vagino-recto-abdominal examination showed a significant improvement in pelvic mobility. The patient’s pain score was markedly reduced to 2 on the VAS. A 6-month postoperative transvaginal ultrasound indicated an AFC of 6–7 in the remaining left ovary.

In March 2024, the patient underwent a natural cycle embryo transfer to a single blastocyst. The serum HCG level was 703 mIU/mL after 11 days. Ultrasound at 30 and 40 days after embryo transfer indicated a normal intrauterine pregnancy (Figures 3C, D). No significant EM symptoms were noted during the pregnancy. However, at 35 weeks and 1 day of gestation, the cesarean section was performed due to fetal growth restriction (3 weeks behind gestational age) secondary to pregnancy-induced hypertension. A healthy male infant was delivered with Apgar scores of 10 at 1 and 5 min.

Written informed consent for publication of clinical details and accompanying images was obtained from both patients. All presented data are anonymized. The publication of non-identifiable ultrasound images and surgical video footage was specifically authorized within the consent process.

## Discussion

This case report illustrates the efficacy of a sequential strategy by combining ART, radical surgery, and natural-cycle frozen embryo transfer (FET) for managing infertility associated with DIE. This strategy not only highlights the necessity for multidisciplinary collaboration (reproductive medicine, gynecological surgery, urology, and colorectal surgery) in managing complex DIE cases but also emphasizes the importance of individualized treatment based on patients’ symptoms, reproductive needs, and the extent of anatomical impairment.

The significant alleviation of DIE-related symptoms postoperatively can be attributed to the synergistic effect of radical surgery and subsequent medical therapy. In both cases, patients received postoperative treatment with a gonadotropin-releasing hormone agonist (GnRH-a) or dienogest, agents proven to suppress residual microscopic disease and reduce the risk of recurrence (10, 11). This comprehensive management strategy, by integrating optimal cytoreductive surgery with adjuvant medical treatment, is consistent with the principles of long-term endometriosis care. It likely played a crucial role in achieving sustained symptomatic relief and creating a favorable pelvic environment for subsequent embryo implantation.

The 2018 Chinese Expert Consensus on Long-Term Management of Endometriosis indicates that surgical intervention does not significantly improve postoperative pregnancy rates in DIE-related infertility; however, it presents limitations due to the risk of surgical trauma and complications (12). Therefore, for DIE with minimal pain symptoms, clinical guidelines recommend IVF-ET as the first-line treatment option, with surgery designated as a remedial approach if there are repeated failures of IVF-ET (12). While the 2022 European Society of Human Reproduction and Embryology Guideline stressed that treatment of DIE-related infertility should be based on the severity of symptoms and patient preferences, it also underlined that the impact of surgery on reproductive outcomes lacks definitive evidence to support its necessity (13). From the current cases, we recommend that the decision for surgical treatment in patients with DIE-related infertility should be individualized. We have adopted a sequential strategy of prioritizing “fertility preservation” to mitigate the potential impact of surgery on ovarian function through preoperative embryo cryopreservation. This approach is particularly applicable to patients with extensive pelvic adhesions or diminished ovarian reserve. However, for asymptomatic patients with DIE-related infertility, current clinical evidence supports prioritizing ART over surgical intervention.

In the present cases, the indication for radical surgery was compelling, driven by the progression of debilitating symptoms and the need to prevent serious complications. In one patient, hydronephrosis constituted an absolute indication for intervention to preserve renal function, a concern that is particularly critical before a planned pregnancy. Moreover, as documented in the literature, untreated bowel DIE carries a quantifiable risk of catastrophic events such as obstruction and perforation, justifying proactive surgical management (14–18). These considerations underscore that for patients with severe symptoms or impending organ dysfunction, surgical intervention remains a cornerstone of comprehensive care.

Notably, in the second case, a laparoscopic myomectomy was performed concurrently with the DIE surgery. Submucosal and certain subserosal leiomyomas are known to adversely affect fertility and pregnancy maintenance (19). Retrospective studies further support their negative impact on clinical pregnancy and live-birth rates (20, 21). Therefore, the myomectomy in this case likely served a complementary role in optimizing the uterine environment for subsequent embryo implantation, following the correction of the pelvic pathology caused by DIE. This underscores the value of a comprehensive surgical strategy that addresses all concomitant pathologies—both endometriotic and uterine—to maximize the potential for a successful reproductive outcome.

Natural cycle FET may reduce the risk of adverse obstetric and neonatal outcomes (22, 23). In this case report, the choice of natural cycle FET was primarily based on the following considerations: Although CA125 is not a specific biomarker for endometriosis, it was used in these cases as a non-specific marker to monitor disease activity and treatment response rather than diagnostic purposes (24), and postoperative normalization of CA125 levels in these two cases might indicate effective inflammation control. Specifically regarding the timing of embryo transfer, we utilized the trend of CA125 as a practical guide. A reduction of CA125 to relatively low levels provided biochemical evidence of a quiescent inflammatory state (24). The improvement of serum CA125 and anatomical restoration by ultrasound and pelvic examination suggests a good prognosis for frozen embryo transfer. The surgery significantly improved the pelvic anatomical environment. Additionally, the natural cycle simulates physiological hormonal fluctuations, potentially mitigating the risk of activating residual lesions from exogenous estrogen stimulation. Current evidence suggests that natural cycles offer potential advantages over artificial cycles, including simplified medication, a lower incidence of pregnancy-related complications (such as preeclampsia), and better neonatal outcomes (25).

## Conclusion

In conclusion, for patients with DIE who present with progressive organ dysfunction, intractable pain, or a history of ART failures, individualized surgical treatment may play a crucial role in optimizing the reproductive microenvironment and improving pregnancy outcomes. The sequential strategy of “freeze-all embryos, perform radical surgery, natural cycle embryo transfer” within ART and surgical sequences showed promising outcomes in severe DIE patients with infertility.

## Data Availability

The original contributions presented in the study are included in the article/Supplementary material, further inquiries can be directed to the corresponding authors.

## Supplementary material

SUPPLEMENTARY FIGURE S1 Endoscopic images of case 1. At 10 cm from the anal verge, the rectum shows mucosal thickening with slight narrowing of the lumen, and the surface appears rough and reddened; rectal mucosal lesions (endometriosis not excluded).
