# Supplementary figures and images for: Case Report: Two clinical cases of severe deep infiltrating endometriosis with infertility—transplantation first or surgery first? Natural cycle or artificial cycle?
Source: Front Med (Lausanne). 2025 Dec 16;12:1725614. doi: 10.3389/fmed.2025.1725614 (PMC12748218; doi:10.3389/fmed.2025.1725614)

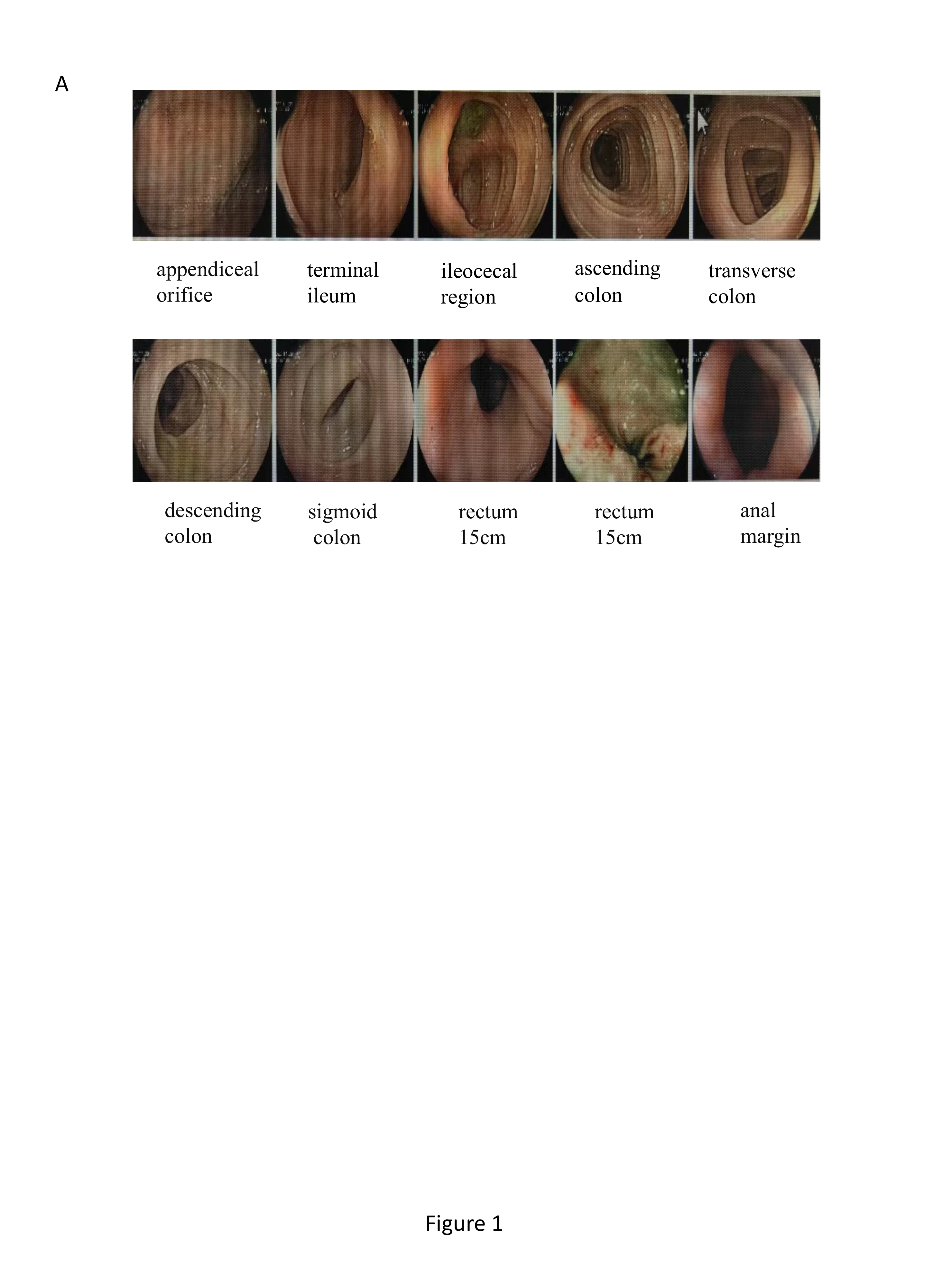

Supplement: SUPPLEMENTARY FIGURE S1 — Endoscopic images of case 1. At 10 cm from the anal verge, the rectum shows mucosal thickening with slight narrowing of the lumen, and the surface appears rough and reddened; rectal mucosal lesions (endometriosis not excluded). [file Image_1.TIF]
