# Supplementary material for: Case Report: Two clinical cases of severe deep infiltrating endometriosis with infertility—transplantation first or surgery first? Natural cycle or artificial cycle?
Source: Front Med (Lausanne). 2025 Dec 16;12:1725614. doi: 10.3389/fmed.2025.1725614 (PMC12748218; doi:10.3389/fmed.2025.1725614)
Supplement: Supplementary file 2 [file Data_Sheet_1.PDF]

CONSENT

I Youdi Fang [PRINT FULL NAME] give my consent for the Material about me/the patient to appear in a Frontiers Media S.A. publication.

I confirm that I: (please tick boxes to confirm)

☑ have seen the photo, image, text or other material about me/the patient

☑ have read the article to be submitted to Frontiers in Medicine

☑ am legally entitled to give this consent.

I understand the following:

(1) The Material will be published without my/the patient's name attached, however I understand that complete anonymity cannot be guaranteed. It is possible that somebody somewhere - for example, somebody who looked after me/the patient or a relative - may recognize me/the patient.

(2) The Material may show or include details of my/the patient's medical condition or injury and any prognosis, treatment or surgery that I have/the patient has, had or may have in the future.

(3) The article may be published in a journal which is distributed worldwide. Frontiers Media.S.A.'s publications go mainly to doctors and other healthcare professionals but are also seen by many others including academics, students and journalists.

(4) The article, including the Material, may be the subject of a press release, and may be linked to from social media and/or used in other promotional activities. Once published, the article will be placed on a Frontiers in Medicine website and may also be available on other websites.

(5) The text of the article will be edited for style, grammar and consistency before publication.

(6) I/the patient will not receive any financial benefit from publication of the article.

(7) I can revoke my consent at any time before publication, but once the article has been committed to publication ("officially published online") it will not be possible to revoke the consent.

(8) This consent form will be securely retained by Frontiers Media S.A. in accordance with the law, for no longer as necessary. All personal data provided herein will be used and retained in compliance with all applicable laws and regulations governing the privacy and security of personal information, including, but not limited to, the General Data Protection Regulation (GDPR) (EU) 2016/679 and other relevant international and

local data protection laws.

Please tick box to confirm the following:

☐ Where this consent relates to an article in Frontiers in Medicine Case Reports, I have/the patient has had the opportunity to comment on the article and I am satisfied that the comments, if any, have been reflected in the article.

Signed: Youdi Fang
Telephone: 86-18755197855

Print name: Youdi Fang
Date: 14/09/2025
